# Pharmacokinetics of menbutone after intravenous and intramuscular administration to sheep

**DOI:** 10.3389/fvets.2022.980818

**Published:** 2022-08-08

**Authors:** Raquel Diez, M. Jose Diez, Juan J. Garcia, Jose M. Rodriguez, Cristina Lopez, Nelida Fernandez, Matilde Sierra, Ana M. Sahagun

**Affiliations:** Department of Biomedical Sciences, Veterinary Faculty, Institute of Biomedicine (IBIOMED), University of Leon, Leon, Spain

**Keywords:** choleretic, intramuscular, intravenous, menbutone, pharmacokinetics, sheep

## Abstract

Menbutone is a drug currently approved in several European Union (EU) countries to treat digestive disorders in different animal species. The objective of this study was to establish the pharmacokinetic parameters resulting from intravenous (IV) and intramuscular (IM) administration of this drug in sheep. Menbutone was administered to 12 animals at the dose of 10 mg/kg for both IV and IM routes. Plasma samples were collected up to 24 h (15 points, IV route; 14 points, IM route). Concentrations were determined using high-performance liquid chromatography with photodiode-array (PDA) detection, following a method validated according to the EMEA/CHMP/EWP/192217/2009 guideline. Pharmacokinetic data were analyzed by non-compartmental methods. After IV administration, a total clearance (Cl) of 63.6 ± 13.6 mL/h/kg, a volume of distribution at steady-state (V_ss_) of 259.6 ± 52.7 mL/kg, and an elimination half-life (t_½λ_) of 6.08 ± 2.48 h were calculated. After IM administration, menbutone peak plasma concentration (C_max_) was 18.8 ± 1.9 μg/mL, the time to reach C_max_ (t_max_) 3.75 ± 0.45 h, the mean absorption time (MAT) 3.31 ± 1.36 h, and the fraction of dose absorbed (F) 103.1 ± 23.0 %. The results obtained indicate that menbutone absorption after IM administration is quick and complete.

## Introduction

Menbutone, or genabilic acid (4-methoxy-γ-oxo-1-naphthalene butanoic acid), is a choleretic drug commonly used in veterinary medicine. This compound increases both bile secretion and peptic and pancreatic juices by 2–4.5 times baseline (1). Its estimulating effect lasts for 2–3 h, although the clinical effect seems to remain longer (1, 2). This drug has been routinely employed for years to treat a wide number of digestive disorders of primary or secondary origin, to restore normal gastrointestinal function when stimulation of digestive secretions is needed in ruminants, horses, pigs or dogs. These dysfunctions are relatively common in animal husbandry, and may result in large economic losses. In recent years, several veterinary medicines containing this active ingredient have been approved for use in most European Union (EU) countries by different procedures (3, 4). On account of its safety, the EU has established that no maximum residue level (MRL) is required for menbutone in food-producing animals (5, 6).

Despite this wide use, basic information regarding menbutone pharmacokinetics for these species is very scarce. The data available are reported in the summary of product characteristics (SPC) of those medicines containing this compound, and no data have been found for sheep. On the other hand, this drug may have beneficial effects on the oral pharmacokinetics of other active ingredients. Menbutone has a choleretic action, and its concomitant use with drugs of high water solubility and reduced oral absorption may be of clinical interest, as it may improve their oral absorption and, consequently, their efficacy. To carry out studies for the development of this kind of associations, it is important to know menbutone pharmacokinetics.

In the present study we have characterized the pharmacokinetics of this drug in sheep after intravenous (IV) and intramuscular (IM) administration, as both routes of administration are used in clinical practice.

## Materials and methods

### Reagents

Menbutone pure reference standard (98.99%) was purchased from LGC-Dr. Ehrenstorfer (LGC Labor GmBH, Ausburg, Germany). Sparfloxacin was used as internal standard (IS) (purity 98%), and supplied from Sigma-Aldrich (Merck, Darmstadt, Germany). Reagents and solvents used for drug extraction and analysis were: Acetonitrile (HiPerSolv Chromanorm^®^, VWR, Radnor, PA, USA), methanol (LiChrosolv, Merck, Madrid, Spain), monopotassium phosphate (AnalaR Normapur^®^, Radnor, PA, USA), sodium hydroxide 1N (Panreac Quimica S.A., Barcelona, Spain) and acetic acid (HiperSolv Chromanorm, VWR, Radnor, PA, USA). HPLC grade water was produced in our laboratory by using a Millipore Milli-Q Gradient water purification system (Waters Corporation, Mildford, MA, USA), and used for the extraction and quantification procedures. For the solid phase extraction (SPE) Oasis HLB 1cc 30 mg cartridges (Waters Corporation, Mildford, MA, USA) were employed. A commercially available formulation (Digestosyva^®^ 100 mg/mL, Laboratorios Syva S.A.U., Leon, Spain) containing menbutone was used for IV and IM administrations to sheep.

### Animals

Twelve clinically healthy adult non-pregnant and non-lactating female Churra sheep, ranging 4–5 years of age and 52–65 kg bodyweight, were used for this study. They were group-housed indoors in the Experimental Farm of the University of Leon (Spain), in an adequately ventilated building (temperature 19 ± 2°C). Sheep were allocated within 15 days before the experiment for acclimation period, and maintained in these conditions until the end of the trial. Animals' health was closely monitored before and throughout the experimental period by a veterinarian. They were fed on a diet of alfalfa hay and pelleted feed concentrate twice a day, and water and saltlick were given *ad libitum*. Procedures and management protocols were approved in advance by the Ethics Committee of the University of Leon and the regional authorities (ULE-007-2020).

### Experimental design

The study had a randomized two-period crossover design with a 2-week washout between treatments. Sheep were divided into two groups of six animals each. Menbutone was used in accordance with the terms of the marketing authorization, at a dose of 10 mg/kg by the intravenous (IV) (group 1) and intramuscular (IM) (group 2) routes. After the 2-week washout period, group 1 received the drug by IM administration, and group 2 by IV injection. For the IV administration, MEN was slowly injected into the left jugular vein slowly, in no <1 min, whereas the intramuscular administration was carried out into the deep gluteal muscle of the right hind limb. The commercially available formulation described before was always used (Digestosyva^®^ 100 mg/mL).

Blood samples were collected in both groups by venipuncture into heparinized tubes (Vacutainer^®^, BD, Plymouth, UK). After IV administration, blood samples were taken from the contralateral jugular vein just before drug administration (time 0) and at 0.25; 0.5; 0.75; 1; 1.25; 1.5; 2, 3, 4, 6, 8, 10, 14 and 24 h. After intramuscular administration, blood samples were drawn just prior injection (time 0) and at 0.5; 0.75; 1; 1.25; 1.5; 2, 3, 4, 6, 8, 10, 14 and 24 h after dosing. Samples were immediately centrifuged at 1,500 rpm for 20 min, and the plasma obtained was stored at −20°C until analysis.

### Analytical procedures

Menbutone extraction from plasma samples was performed according to a method previously validated following the EMA guideline EMEA/CHMP/EWP/192217/2009 (7, 8). Analysis was carried out by high-performance liquid chromatography (HPLC) with photodiode array detector (PDA model 2998) in a Waters Alliance e2695 system (Waters Corporation, Mildford, MA, USA).

Plasma samples were processed according to a previously published method (7). Solid-phase extraction (SPE) with Oasis HLB 1 cc 30 mg cartridges (Waters Corporation, Mildford, MA, USA) was used. In brief, 1 mL plasma was spiked with the internal standard (IS) (sparfloxacin, 20 μg/mL), and deproteinized with 1 mL 10% acetic acid, shaken for 1 min and centrifuged at 1,620 g for 10 min. Supernatant was then transferred into the SPE cartridges (previously conditioned with 1 mL methanol and 1 mL HPLC grade water). After having washed them twice with 1 mL HPLC grade water, cartridges were properly dried and eluted with 1 mL mobile phase. 20 μL of eluate was injected into the HPLC system. All procedures were performed at room temperature.

Chromatographic separation was achieved on an Xbridge BEH C_18_ column (4.6 × 250 mm, 5 μm) (Waters Corporation, Milford, MA, USA), using an isocratic mobile phase consisting of acetonitrile and monopotassium phosphate buffer (1.36 g/L) 49:51 (v/v), at a flow rate of 1.2 mL/min. The wavelength was set at 236 and 297 nm. Under these conditions, the retention times were 2.2 min for the IS and 4.5 min for menbutone. The lower limit of quantification (LLOQ) was 0.2 μg/mL and the limit of detection (LOD) 0.08 μg/mL. The mean recovery of menbutone was 91.12 ± 9.25%. The study was conducted under the Good Laboratory Practice (GLP) regulations at our GLP-compliant laboratory LAFARLE (University of Leon, Spain), certified by the Spanish Agency of Medicines and Medical Devices (9).

### Pharmacokinetic analysis

Non-compartmental pharmacokinetic analysis of menbutone in plasma was performed for each sheep using a commercially available software (Phoenix WinNonlin, version 8.3; Certara Inc., St. Louis, MO, USA). Analysis was performed from the raw data, with expressions based on statistical moments theory (10) and standard formulae (11, 12). Plasma elimination rate constant (λ) was estimated by least squares regression of the logarithm of plasma concentration vs. time curve over the terminal elimination phase, and t_1/2λ_ as 0.693/λ. AUC and AUMC were calculated by the trapezoidal rule from the time of treatment administration to the last measurable concentration, and extrapolated to infinity by dividing the last experimental concentration by the terminal slope (λ). MRT was calculated as AUMC/AUC. Mean absorption time (MAT) was obtained as MRT_IM_-MRT_IV_. Maximum plasma concentration (C_max_) and the time to reach C_max_ (t_max_) were determined directly from the plasma concentration-time curves.

The fraction of dose absorbed (F) was calculated as


$$\begin{array}{l} F=\;\frac{AUC_{IM}}{AUC_{IV}}\cdot 100 \end{array}$$

where AUC_IV_ and AUC_IM_ are the area under the curve after IV and IM administration, respectively.

### Statistical analysis

Pharmacokinetic parameters were calculated for each animal and reported as mean ± standard deviation (SD). The statistical analysis was performed using the IBM SPSS for Windows software package v. 26 (IBM Corporation, Armonk, NY, USA). Data were tested for normality using the Shapiro-Wilk test. If data were normal, they were compared using a paired *t*-test; if not, a Wilcoxon signed-rank test was used. A value of p ≤ 0.05 was considered significant.

## Results

**Figure 1:**
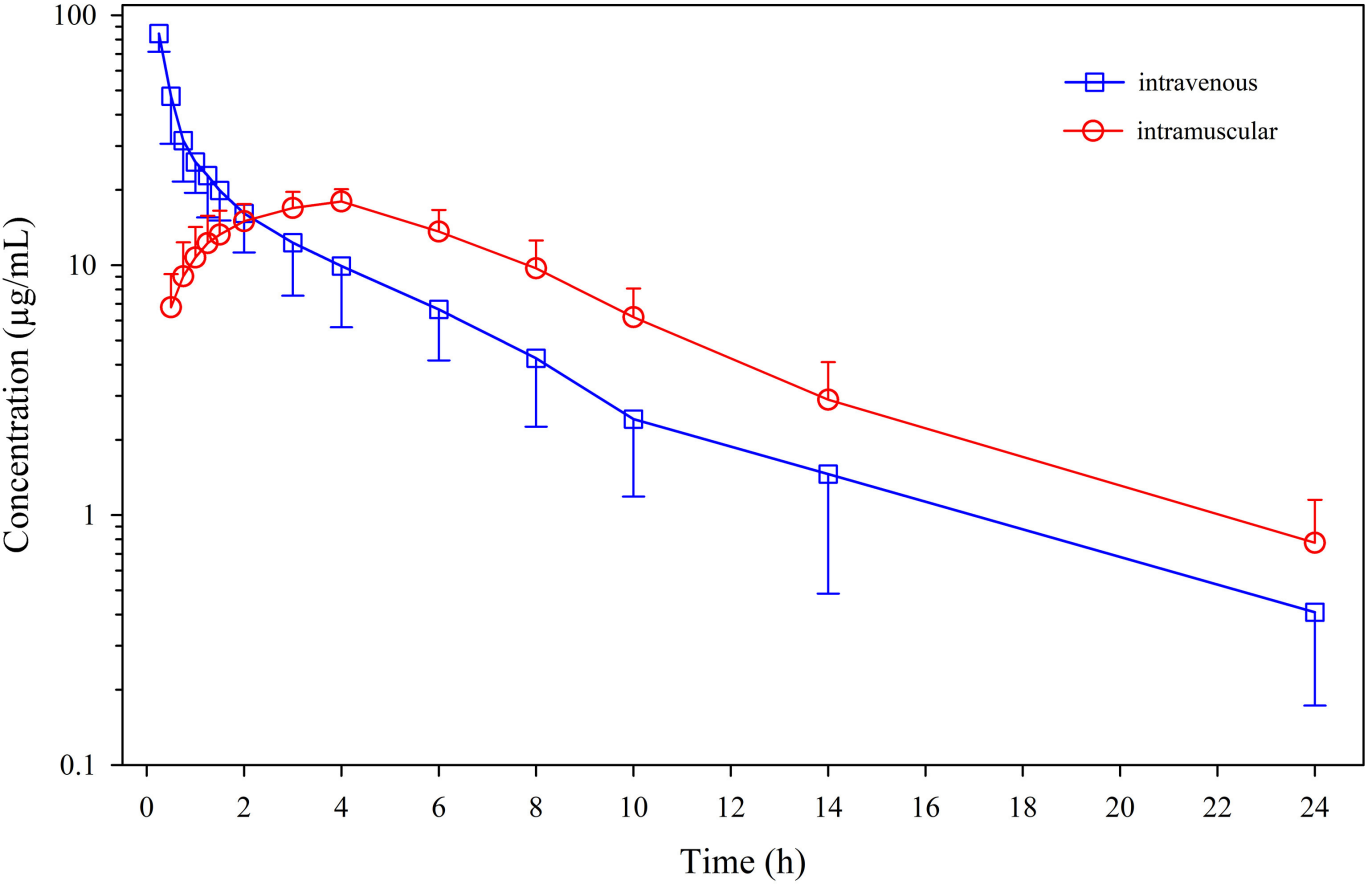
Plasma concentrations (mean ± SD) of menbutone obtained after intravenous and intramuscular administration (10 mg/kg) to 12 sheep.

Mean ± SD plasma concentrations of menbutone as a function of time after IV and IM administrations are shown in Figure 1. Individual data are included in Supplementary Tables S1, S2, Supplementary Figure S1. The drug was present in detectable concentrations at all collection times following IV and IM administration. Non-compartmental pharmacokinetic parameters after IV administration are summarized in Table 1. Plasma concentrations declined rapidly, with a terminal half-life (t_½λ_) of 6.08 ± 2.48 h and a mean residence time (MRT_0−∞_) of 4.23 ± 1.18 h. This drug exhibits a high total body clearance (Cl) (63.6 ± 13.6 mL/kg/h) with a volume of distribution at steady-state (V_ss_) of 259.6 ± 52.7 mL/kg. An AUC_0−∞_ of 165.0 ± 40.1 μg·h/mL was also calculated.

**Table 1:** Non-compartmental pharmacokinetic parameters (mean ± SD) of menbutone obtained after IV and IM administration (10 mg/kg) to 12 sheep.

**Pharmacokinetic parameter**	**Unit**	**IV**	**IM**
λ	h^−1^	0.13 ± 0.04	0.15 ± 0.03^c^
t_1/2λ_	h	6.08 ± 2.48	4.88 ± 1.18^c^
C_max_	μg/mL		18.8 ± 1.9
t_max_	h		3.75 ± 0.45
V_ss_	mL/kg	259.6 ± 52.7	
V_z_	mL/kg	550.5 ± 219.7	
V_z_/F	mL/kg		431.4 ± 107.6^d^
Cl	mL/kg/h	63.6 ± 13.6	
Cl/F	mL/kg/h		61.8 ± 10.4^e^
AUC_last_	μg·h/mL	160.9 ± 38.7	160.1 ± 25.1^c^
AUC_0−∞_	μg·h/mL	165.0 ± 40.1	166.0 ± 27.4^c^
AUMC_last_	μg·h^2^/mL	575.0 ± 225.1	1,079.7 ± 252.6^a^
AUMC_0−∞_	μg·h^2^/mL	721.8 ± 321.7	1,269.2 ± 348.3^a^
MRT_last_	h	3.48 ± 0.66	6.68 ± 0.63^a^
MRT_0−∞_	h	4.23 ± 1.18	7.54 ± 0.93^b^
MAT_last_	h		3.20 ± 0.64
MAT_0−∞_	h		3.31 ± 1.36
F	%		103.1 ± 23.0

λ, slope of terminal phase; t_1/2λ_, half-life associated with λ; C_max_, maximum plasma concentration; t_max_, time to reach C_max_; V_ss_, volume of distribution at steady-state; V_z_, apparent volume of distribution calculated by the area method; V_z_/F, apparent volume of distribution; Cl, total clearance; Cl/F, apparent clearance; AUC_last_, area under the plasma concentration-time curve from cero to last collected time point; AUC_0−∞_, area under the plasma concentration-time curve from cero to infinity; AUMC, area under the first moment curve; MRT, mean residence time; MAT, mean absorption time; F, fraction of dose absorbed.

a Significantly different from IV parameter (t test, p ≤ 0.05);

b Significantly different from IV parameter (Wilcoxon signed-rank test, p ≤ 0.05);

c No significant differences with IV parameter;

d No significant differences with V_z_;

e No significant differences with Cl.

Table 1 also includes non-compartmental parameters obtained after IM administration. A C_max_ of 18.8 ± 1.9 μg/mL was reached at 3.75 ± 0.45 h (t_max_), and the AUC_0−∞_ was slightly higher (166.0 ± 27.4) μg·h/mL than that obtained by the IV route. The value calculated for F was 103.1 ± 23.0 %, indicating a total absorption. Total mean absorption time (MAT_0−∞_) was 3.31 ± 1.36 h. Significant differences were observed for MRT_0−∞_ (Wilcoxon signed-rank test), and AUMC_last_, AUMC_0−∞_ and MRT_last_ (paired *t*-test) when parameters from both routes of administration (IV and IM) were compared. No significant differences were found for λ, t_1/2λ_, AUC_last_, AUC_0−∞_, V_z_-V_z_/F, Cl-Cl/F.

One of 12 sheep fell down after IV administration, but recovered immediately, and 4 of the animals showed itching at the IM injection site just at the moment of the administration. No more other adverse reactions were observed during the study in the animals treated.

## Discussion

To the best of our knowledge, this is the first study on the pharmacokinetics of menbutone after IV and IM administration in sheep. As explained before, no data on the pharmacokinetics of this compound has been found in the scientific literature in any of the domestic animal species for which this treatment is indicated. In fact, only few data are available for some pharmacokinetic parameters in the SPC of those medicinal products approved in the EU, as the holders of the marketing authorization are not obliged to make public those studies carried out to obtain this authorization. Several papers reported methods to determine menbutone residues in different tissues (7, 13, 14), and only one study has described in a preliminary way the distribution, metabolism and excretion of menbutone in rats (2).

Digestive diseases are common in veterinary practice, and anorexia, gastroenteritis, constipation or indigestion are present in many animals, as concomitant or secondary diseases. Menbutone is an interesting therapeutic option to reverse or alleviate them, which would explain its wide use in various animal species in many EU countries.

After IV administration, menbutone shows a wide distribution in the body. We have also observed that plasma concentrations fell rapidly. The initial steep decline in plasma concentrations was probably due to the high clearance showed by menbutone. For this drug, the overall body extraction rate (E), calculated as Cl/Q_c_, where Q_c_ is the cardiac output, and can be estimated as *Qc* = 180·*BW*(*kg*)^−0.19^ (15), would be 0.762 during a single passage through the clearing organ for a sheep with mean weight of 57 kg, a value next to 1, the maximum for E. This fast disappearance from the body would be in coherence with an excretion of 51–58% radiolabeled menbutone in urine and feces after 6 h and 83% after 24 h when the drug was administered orally to rats (2).

In most animal species both routes of administration (IV and IM) are used for this choleretic drug. In our study the values obtained for C_max_, t_max_, MAT and F indicate that menbutone absorption after IM administration is complete and with a high rate, and it is also observed that absorption is quicker than elimination. On the other hand, higher final plasma concentrations have been reached after the IM administration, which would also account for maintaining its choleretic action. Therefore, in this animal species the IM administration is an easy and feasible treatment option. Moreover, the only adverse reaction observed by the IM route was itching, which affected one third of the animals.

A great variability in plasma concentrations and pharmacokinetic parameters has also been observed among animals. As it is the first study in which the pharmacokinetic behavior of this compound has been defined, it is difficult to correlate this variability with any factor. Regarding the IV route, the enterohepatic circulation (2) of the drug may have influenced this variability, whereas for the IM administration, differences in the absorption rate from the injection site should also be taken into account.

Drug-drug interactions are of growing interest in veterinary medicine due their possibilities of improving the efficacy and safety of those treatments already available. The choleretic properties of menbutone, increasing bile secretion to intestine, may be used to favor the absorption of compounds with poor water solubility. For instance, it has been shown that this drug has significantly augmented the absorption of the anthelmintic metabolite albendazole sulphoxide from the intestinal lumen in sheep (16). Menbutone would act in a similar way as a fatty meal in healthy human subjects. In this way, Ochoa et al. (17) reported an increase in the oral bioavailability of albendazole due to a higher dissolution of this drug in the gastrointestinal tract by stimulating bile secretion.

A basic knowledge of drug pharmacokinetics is essential to ensure a rational use of compounds in target species (18). The current study contributes reliable data on the IV and IM pharmacokinetics of menbutone. The results showed that after IV injection, menbutone plasma concentrations exhibits a fast elimination from the body. After IM administration drug absorption is complete. A wide individual variation has also been observed. Further studies are needed to characterize menbutone pharmacokinetics in other animal species, as well as to assess those potentially beneficial drug interactions that may result.

## Data availability statement

The original contributions presented in the study are included in the article/Supplementary material. Further inquiries can be directed to the corresponding author.

## Ethics statement

The animal study was reviewed and approved by Ethics Committee of the University of Leon and regional authorities.

## Author contributions

MS and JJG designed the study. JMR, NFM, CL, and AMS carried out the animal work. RD and MJD completed the laboratory analysis. MJD performed the calculations. MJD, JJG, RD, and NFM carried out the data analysis. MS, JJG, NFM, and AMS wrote the manuscript. All authors contributed to the article and approved the submitted version.

## Conflict of interest

The authors declare that the research was conducted in the absence of any commercial or financial relationships that could be construed as a potential conflict of interest.

## Publisher's note

All claims expressed in this article are solely those of the authors and do not necessarily represent those of their affiliated organizations, or those of the publisher, the editors and the reviewers. Any product that may be evaluated in this article, or claim that may be made by its manufacturer, is not guaranteed or endorsed by the publisher.
