# Supplementary material for: Pharmacokinetics of menbutone after intravenous and intramuscular administration to sheep
Source: Front Vet Sci. 2022 Aug 8;9:980818. doi: 10.3389/fvets.2022.980818 (PMC9393588; doi:10.3389/fvets.2022.980818)

Supplementary Material

**TABLE S1.** Individual and mean ± SD plasma concentrations of menbutone obtained after intravenous administration (10 mg/kg) to 12 sheep.

| **Time (h)** | **Animals** | | | | | | | | | | | | **Mean ± SD** |
| --- | --- | --- | --- | --- | --- | --- | --- | --- | --- | --- | --- | --- | --- |
|  | **1** | **2** | **3** | **4** | **5** | **6** | **7** | **8** | **9** | **10** | **11** | **12** |  |
| 0.25 | 85.608 | 102.766 | 80.832 | 104.938 | 73.524 | 69.530 | 92.297 | 98.715 | 84.049 | 69.770 | 68.282 | 83.616 | 84.494 ± 12.984 |
| 0.5 | 51.296 | 62.451 | 36.251 | 67.250 | 28.919 | 34.945 | 80.048 | 42.957 | 61.276 | 32.627 | 30.118 | 40.831 | 47.414 ± 16.774 |
| 0.75 | 27.582 | 18.626 | 32.374 | 24.584 | 26.161 | 34.480 | 58.079 | 36.144 | 28.433 | 32.539 | 24.002 | 35.243 | 31.521 ± 9.889 |
| 1 | 24.295 | 17.567 | 24.274 | 19.235 | 23.153 | 26.940 | 41.596 | 33.435 | 24.021 | 28.229 | 23.526 | 24.072 | 25.862 ± 6.387 |
| 1.25 | 18.072 | 16.907 | 19.473 | 17.372 | 21.223 | 26.764 | 40.852 | 32.850 | 21.707 | 22.137 | 19.357 | 17.228 | 22.828 ± 7.306 |
| 1.5 | 17.736 | 15.447 | 16.794 | 16.732 | 17.617 | 23.318 | 25.754 | 31.504 | 21.155 | 18.016 | 18.654 | 15.866 | 19.883 ± 4.795 |
| 2 | 14.541 | 15.243 | 9.113 | 16.021 | 10.571 | 16.625 | 21.351 | 27.958 | 15.011 | 15.827 | 17.425 | 13.592 | 16.106 ± 4.865 |
| 3 | 12.751 | 9.761 | 5.392 | 13.314 | 8.021 | 11.178 | 19.756 | 21.756 | 11.170 | 15.562 | 9.097 | 9.849 | 12.300 ± 4.750 |
| 4 | 10.294 | 8.088 | 4.876 | 11.050 | 7.867 | 4.811 | 17.330 | 18.247 | 10.091 | 11.681 | 7.144 | 7.568 | 9.921 ± 4.276 |
| 6 | 5.791 | 8.418 | 3.648 | 6.058 | 5.209 | 4.495 | 10.751 | 11.035 | 7.495 | 8.062 | 4.607 | 4.289 | 6.655 ± 2.494 |
| 8 | 4.490 | 5.266 | 2.603 | 2.539 | 4.333 | 1.774 | 6.186 | 8.543 | 5.970 | 3.930 | 2.807 | 2.469 | 4.242 ± 1.985 |
| 10 | 2.464 | 4.257 | 1.574 | 1.971 | 1.462 | 0.821 | 2.374 | 5.328 | 2.567 | 2.452 | 2.053 | 1.749 | 2.423 ± 1.237 |
| 14 | 1.404 | 4.037 | 1.163 | 1.618 | 0.813 | 0.473 | 1.327 | 2.494 | 1.614 | 1.058 | 0.899 | 0.618 | 1.460 ± 0.975 |
| 24 | 0.423 | 0.726 | 0.226 | 0.912 | 0.207 | 0.238 | 0.650 | 0.395 | 0.425 | 0.264 | 0.217 | 0.220 | 0.408 ± 0.235 |

*SD: standard deviation.*

**TABLE S2.** Individual and mean ± SD plasma concentrations of menbutone obtained after intramuscular administration (10 mg/kg) to 12 sheep.

| **Time (h)** | **Animals** | | | | | | | | | | | | **Mean ± SD** |
| --- | --- | --- | --- | --- | --- | --- | --- | --- | --- | --- | --- | --- | --- |
|  | **1** | **2** | **3** | **4** | **5** | **6** | **7** | **8** | **9** | **10** | **11** | **12** |  |
| 0.5 | 2.821 | 3.973 | 7.080 | 2.837 | 10.242 | 8.533 | 5.801 | 7.539 | 7.184 | 8.067 | 8.946 | 8.376 | 6.783 ± 2.428 |
| 0.75 | 4.285 | 4.019 | 9.546 | 4.051 | 12.541 | 10.551 | 10.522 | 9.160 | 8.282 | 14.049 | 10.615 | 10.779 | 9.033 ± 3.315 |
| 1 | 5.473 | 5.493 | 10.665 | 6.563 | 14.956 | 11.189 | 12.602 | 12.045 | 9.890 | 16.563 | 11.914 | 11.468 | 10.735 ± 3.470 |
| 1.25 | 8.456 | 6.609 | 11.568 | 7.827 | 15.458 | 11.214 | 15.511 | 13.663 | 11.256 | 18.032 | 11.904 | 15.562 | 12.255 ± 3.513 |
| 1.5 | 10.451 | 8.547 | 12.353 | 8.159 | 16.398 | 11.950 | 16.186 | 14.457 | 12.139 | 18.446 | 13.205 | 16.658 | 13.246 ± 3.278 |
| 2 | 11.264 | 12.197 | 16.395 | 11.924 | 16.513 | 13.311 | 17.222 | 17.566 | 13.302 | 18.508 | 14.273 | 17.580 | 15.005 ± 2.563 |
| 3 | 13.605 | 15.029 | 16.472 | 12.963 | 19.937 | 16.358 | 18.032 | 19.789 | 16.314 | 20.583 | 14.146 | 20.042 | 16.939 ± 2.706 |
| 4 | 16.200 | 17.732 | 19.056 | 16.394 | 16.893 | 14.465 | 18.866 | 22.024 | 19.999 | 16.339 | 17.611 | 20.334 | 17.993 ± 2.132 |
| 6 | 12.749 | 16.648 | 12.105 | 15.803 | 6.837 | 9.280 | 15.435 | 15.285 | 15.966 | 13.118 | 14.997 | 15.311 | 13.628 ± 2.991 |
| 8 | 9.084 | 13.169 | 7.361 | 13.977 | 4.909 | 6.424 | 7.328 | 10.229 | 12.733 | 9.612 | 9.985 | 11.743 | 9.713 ± 2.848 |
| 10 | 5.495 | 9.691 | 5.168 | 6.944 | 3.913 | 4.305 | 3.442 | 6.833 | 7.339 | 6.560 | 5.952 | 8.663 | 6.192 ± 1.875 |
| 14 | 2.233 | 3.677 | 1.542 | 2.233 | 2.321 | 1.673 | 1.481 | 2.520 | 4.738 | 4.567 | 3.457 | 4.310 | 2.896 ± 1.198 |
| 24 | 0.476 | 0.585 | 0.389 | 0.596 | 0.659 | 0.429 | 0.945 | 0.693 | 1.278 | 1.689 | 0.777 | 0.784 | 0.775 ± 0.377 |

*SD: standard deviation.*

FIGURE S1. Individual plasma concentrations of menbutone obtained after intravenous (🞎) and intramuscular (⭘) administration (10 mg/kg) to 12 sheep
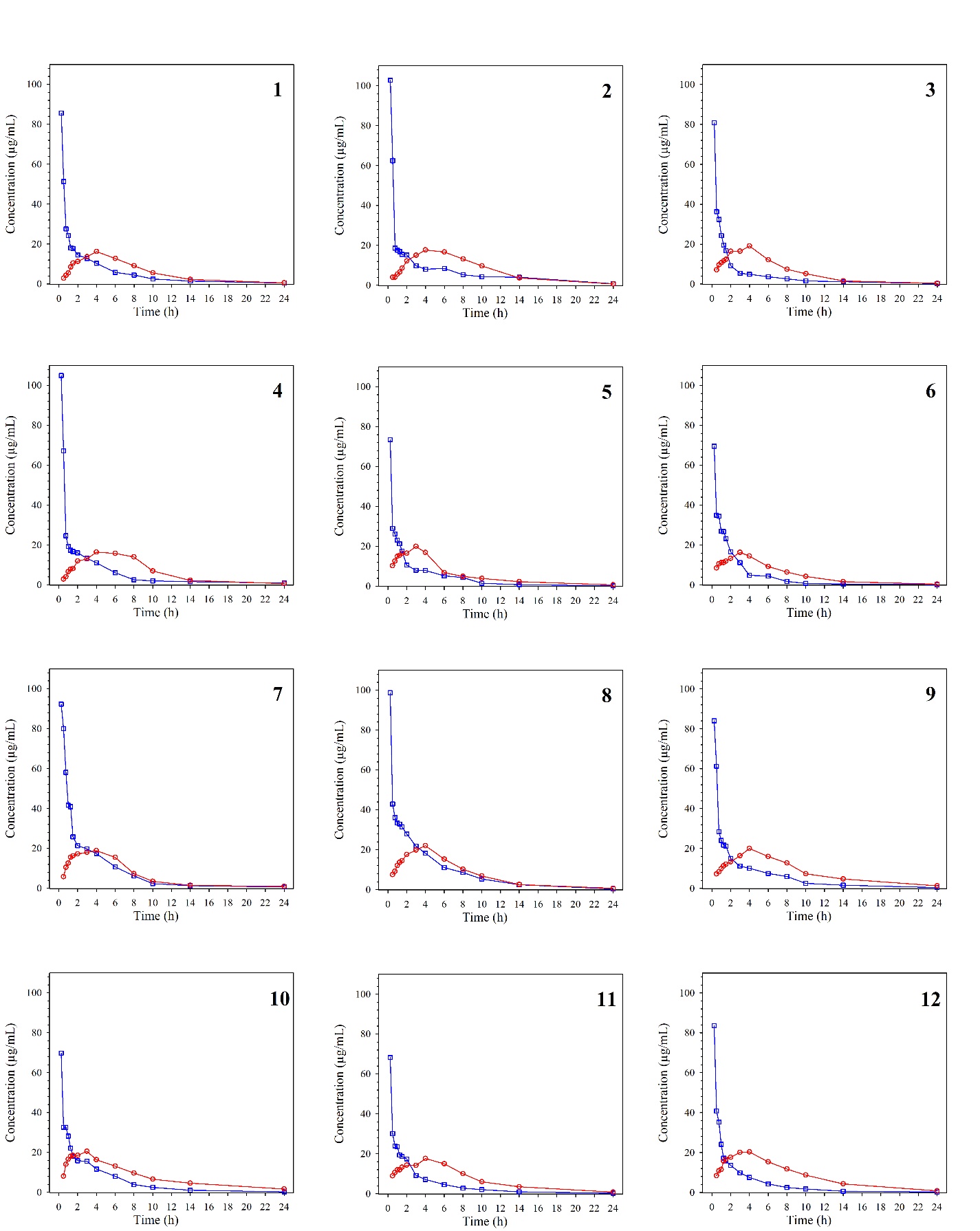

Supplement: Supplementary file 1 [file Data_Sheet_1.docx]
